# Polyacrylonitrile Nanofiber Mats Produced by Solution Blow Spinning: Influence of Process Parameters on Fiber Diameter and Residual Solvent Content

**DOI:** 10.3390/polym18010100

**Published:** 2025-12-29

**Authors:** Natalia Menshutina, Danil Kunaev, Andrey Abramov, Alekseev Aleksandr

**Affiliations:** Department of Chemical and Pharmaceutical Engineering, Mendeleev University of Chemical Technology of Russia, Miusskaya pl. 9, 125047 Moscow, Russia; chemcom@muctr.ru (N.M.); kunaev.d.a@muctr.ru (D.K.); alekseev.a.a@muctr.ru (A.A.)

**Keywords:** solution blow spinning (SBS), polyacrylonitrile (PAN), polymer nanofibers, fiber diameter, residual solvent content, regression analysis, process parameters

## Abstract

This study reports on the fabrication of polyacrylonitrile (PAN) nanofiber mats by solution blow spinning. A fabrication protocol is presented together with a comprehensive investigation of how key process parameters (polymer solution concentration, air pressure, and solution flow rate) affect the residual solvent content and the diameter of the resulting nanofibers. The following dependencies were identified: increasing solution concentration leads to larger fiber diameters, whereas increasing air pressure and decreasing solution flow rate both result in smaller diameters. The residual solvent content exhibits a non-linear dependence on the process parameters with an expressed minimum. The number-average diameter of the nanofibers ranges from 428 to 221 nm. Regression analysis confirmed the statistical significance of the effects of the studied factors on fiber diameter, and the fact that the calculated value of the Fisher criterion is lower than the critical tabulated value indicates that the proposed model is adequate. The determination coefficient of 0.85 demonstrates a high degree of consistency between the model and the experimental data.

## 1. Introduction

**Figure 1:**
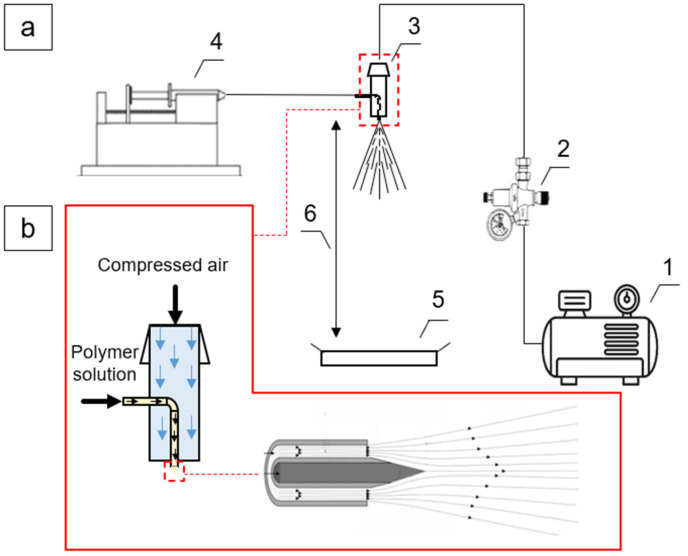
Solution blow spinning (SBS) setup: (**a**) schematic diagram of the device: 1—compressed gas source; 2—pressure regulator; 3—coaxial nozzle; 4—syringe pump; 5—fiber collector; 6—working distance; (**b**) design of the coaxial nozzle and principle of operation.

Solution blow spinning (SBS) is one of the most promising technologies for the production of nanofibers with unique physicochemical and structural characteristics [1]. The process starts with the preparation of a spinning solution, that is, a polymer dissolved in a volatile solvent. The solution is then fed through a coaxial nozzle, where it is exposed to a high-velocity gas stream (Figure 1a). As the gas passes through the outer orifice, its velocity increases, which, according to Bernoulli’s principle, leads to the formation of a low-pressure region at the exit of the inner nozzle [2]. As a result, the solution forms a conical meniscus, known as a Taylor cone, which represents a stable flow regime prior to further jet elongation (Figure 1b).

Under the influence of aerodynamic forces, the solution jet is stretched and thinned, while viscosity and polymer concentration, surface tension, and solvent evaporation rate play key roles. A high gas-flow velocity enhances the extensional forces exerted on the jet, thereby promoting the formation of thin fibers with tailored structural parameters [3]. The physicochemical properties of the spinning solution determine its deformability and the ability to stabilize the fiber shape after solvent evaporation, which is critical for achieving the desired characteristics of the final material [4].

In recent years, considerable attention has been paid to studies focused on the production of polymer nanofibers based on polyacrylonitrile (PAN). PAN nanofibers exhibit a unique combination of properties, including a high specific surface area, porosity, mechanical strength, chemical stability, and the possibility of surface functionalization [5]. These characteristics make PAN-based nanofibers particularly attractive for applications in biomedicine [6,7] and pharmaceutics [8,9].

In biomedicine, polyacrylonitrile (PAN) nanofibers have been widely investigated as scaffolds for tissue engineering because their fibrous morphology can closely mimic the architecture of the extracellular matrix and support cell adhesion, proliferation, and lineage-specific differentiation [10]. In the pharmaceutical field, PAN-based nanofibrous mats are increasingly explored as drug-delivery platforms that enable high drug loading and sustained, and in some cases locally targeted, release of bioactive compounds [11]. Beyond biomedicine and pharmaceutics, PAN nanofibers and their carbonized derivatives represent attractive candidates for a broad range of high-tech applications, including filtration, catalysis, and energy-related systems [12]. In particular, in the energy sector, PAN-derived nanofibrous membranes are used in the development and optimization of high-performance separation media [13], separators for rechargeable batteries [14], and electrode and gas-diffusion components for fuel cells, where a large specific surface area, tunable porosity, and high selectivity are essential. Owing to their small pore size and high specific surface area, polymer nanofibers based on PAN have also been widely used as efficient filtering media capable of capturing fine particulate matter such as aerosols, dust, soot, bacteria, viruses, and allergens. In the manufacture of protective masks and respirators, PAN nanofibers can provide a high level of particle removal efficiency at relatively low breathing resistance, which is advantageous for prolonged wear. Furthermore, surface functionalization of PAN nanofibers with antibacterial and antiviral agents has been shown to further enhance filtration performance and to inhibit the growth of microorganisms on the filter surface [15].

A large number of experimental studies [16,17,18] have analyzed how processing parameters control the diameter and morphology of nanofibers obtained by solution blow spinning and electrospinning. For PAN and other polymers, it has been shown that increasing polymer concentration or solution viscosity generally leads to thicker fibers and a reduced amount of bead-on-string defects, whereas adjusting the driving force (applied voltage in electrospinning or gas pressure in SBS), solution flow rate and collector distance allows the mean diameter and width of the diameter distribution to be tuned within a broad range [19,20]. In the case of PAN/DMF systems, several works have reported that fiber diameter increases with PAN mass fraction and that an optimal processing window exists in which continuous, defect-poor fibers can be obtained, while outside this window jet break-up or excessive thickening occurs [21]. These studies underline the strong coupling between solution properties, process parameters and the resulting fiber dimensions in systems closely related to those considered in the present work.

To increase the efficiency of research aimed at developing new materials with tailored properties and to improve the accuracy of analyzing and predicting the dimensions of the resulting nanofibers, it is reasonable to use regression analysis [22]. This method is based on constructing a mathematical model that describes how changes in the independent variables affect changes in the dependent variable [23]. The main objective of regression analysis is to predict or explain the value of the dependent variable on the basis of the considered factors [24].

In scientific research, regression analysis is widely used to identify and quantify relationships in various fields, ranging from economics and medicine to engineering and the social sciences. For example, in medicine [25], regression helps to assess the influence of risk factors on disease probability; in economics [26], it is used to forecast market trends; and in engineering, it is applied to optimize technological processes [27]. For nanofiber fabrication processes, design-of-experiments and response surface methodology have been applied to quantify the influence of solution concentration, flow rate, driving force and geometrical parameters on electrospun fiber diameter and to build predictive models with high coefficients of determination [28]. However, regression models specifically developed for SBS, and, in particular, for PAN nanofibers produced from DMF solutions, remain comparatively rare, and the combined analysis of fiber diameter with additional process outputs such as residual solvent content has received much less attention [29,30].

In this study, a comprehensive approach to the fabrication of polyacrylonitrile nanofibers by solution blow spinning is presented in order to evaluate and choose the technological process parameters that ensure high functional characteristics of the resulting material. The main objectives of the study are as follows: to carry out a series of experiments with variation in the process parameters (polymer concentration of 13–15 wt.%, air pressure of 0.2–2.5 bar, and solution flow rate of 0.1–1 mL/h); to perform analytical characterization of the obtained materials using scanning electron microscopy; and to construct a regression model to assess the significance of the factors and to predict nanofiber diameters.

## 2. Materials and Methods

### 2.1. Materials and Reagents Used

In this study, polyacrylonitrile (PAN) was used as the polymer for nanofiber production. PAN is a synthetic linear polymer obtained by free-radical polymerization of acrylonitrile monomer. The PAN employed here (Mn ≈ 50 kDa, Hebei Chuanghai Biotechnology Co., Ltd., Shijiazhuang, China) was purchased commercially and used as received without further polymerization.

N,N-Dimethylformamide (DMF) was used as the solvent. DMF is a colorless, viscous liquid with a faint characteristic smell. It has a high dielectric constant and a moderate volatility, which makes it suitable for the solution blow spinning process. The use of more volatile solvents in solution blow spinning may lead to premature solidification of the polymer inside the nozzle and clogging, which is highly undesirable. It should be noted that DMF belongs to hazard class 3 according to the UN classification, which implies relatively high toxicity and the need to comply with strict safety measures during handling. Consequently, the use of DMF limits the applicability of the obtained products in biomedicine and pharmaceutics because of toxicity risks. However, in future studies, it is planned to select and employ safer solvents, which will reduce health risks and allow the production of materials suitable for medical and biological applications.

### 2.2. Development of SBS Installation

**Figure 2:**
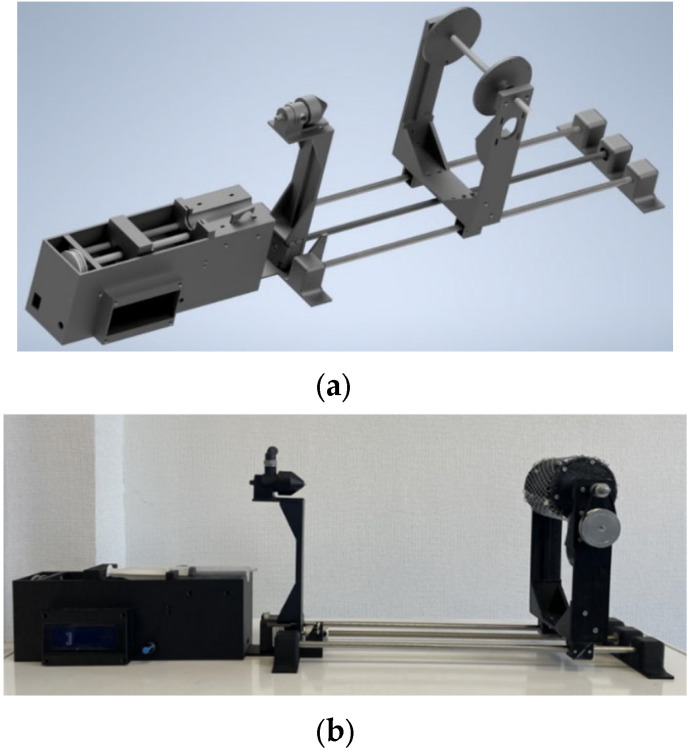
Design of the laboratory setup for solution blow spinning: (**a**)—three-dimensional model of the assembled setup; (**b**)—assembled laboratory installation.

To produce PAN-based nanofibers, a laboratory-scale setup for the solution blow spinning process was developed. The development proceeded in three main stages: (1) creation and assembly of three-dimensional CAD models of each component of the setup (Figure 2a); (2) fabrication of the designed model using additive manufacturing technologies (Figure 2b); and (3) development and implementation of a control system for the designed setup.

The laboratory setup for solution blow spinning (Figure 2) is a modular system comprising three main functional zones: a dosing zone, a nanofiber formation zone, and a collection zone for the resulting nonwoven material. The dosing zone is implemented as a syringe pump designed for use with 20 mL medical syringes. The case also accommodates the process control unit that regulates the process parameters (solution flow rate, collector rotation speed, and collection distance) and displays information for the operator on an LCD screen. An incremental encoder mounted on the front panel of the dosing unit is used to navigate the menu and set the required parameters. For accurate positioning and control of the rotational speed of the mechanical elements, stepper motors driven in microstepping mode are employed.

**Figure 3:**
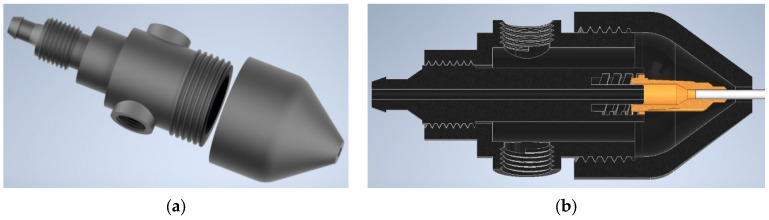
Three-dimensional model of the pneumatic nozzle: (**a**)—exploded view; (**b**)—half-section of the nozzle design.

The next functional zone of the laboratory setup is the nanofiber formation zone, which is implemented using a pneumatic nozzle (Figure 3). The designed nozzle is a modular system that allows the diameters of the outlets for the liquid and gas phases to be varied (Figure 3a).

To vary the diameter of the outlet orifice for the polymer solution, a LUER-LOCK connection system is used. The outlet diameter for the gas phase is adjusted by means of a removable tip with a G3/4″ thread. For compressed gas supply, two ports with internal G1/8″ threads are provided to connect collet fittings for hoses with an inner diameter of 6 mm. Liquid crystal stereolithography was employed to fabricate the nozzle modules, which enable variation in the process parameters over a wide range with a very small step of 22 μm (the resolution of the Phrozen 8K mini printer (Phrozen Tech Co., Ltd., Hsinchu City, Taiwan)). The high precision of this additive manufacturing method significantly expands the experimental parameter space of the process.

The final functional zone of the setup is a rotating collector assembly for fiber deposition, which allows the collection distance during the process to be varied. The distance is adjusted by a trapezoidal screw with a traveling nut driven by a stepper motor. The length of the working zone is limited only by the length of the shafts and the screw; in the present study, the maximum working distance was 50 cm. The collector consists of two plates mounted on an M8 threaded rod and fixed with four nuts. The modular design allows the collector geometry to be varied for different applications.

### 2.3. Preparation of Polymer Solution

PAN solutions with concentrations of 13, 14 and 15 wt.% were prepared by dissolving the appropriate mass of PAN in N,N-dimethylformamide (DMF). The required amount of PAN was added to DMF under magnetic stirring at 300 rpm at room temperature (20 °C) and stirred for 24 h until a homogeneous solution without visible undissolved particles was obtained. The beakers were covered with parafilm to minimize solvent evaporation during mixing. Before loading into the syringes for solution blow spinning, the solutions were allowed to stand for 30 min to release trapped air bubbles. All solutions were used within 24 h after preparation.

### 2.4. Experimental Study of Solution Blow Spinning Process

To investigate the influence of technological parameters on the structure of the final nanofiber-based materials, a full factorial experimental design was used. The following process parameters were varied: polymer solution concentration, air pressure, and solution flow rate.

All experiments were conducted under laboratory ambient conditions. The air temperature during spinning was 20 °C and the relative humidity was 45–55%, as monitored with a calibrated digital thermo-hygrometer. Although no dedicated environmental chamber was used, the variation in these parameters remained within the stated range for all experiments.

The solution flow rate affects both jet stability and fiber dimensions. Too low flow rates can lead to jet instability, whereas excessively high flow rates may result in nozzle clogging. Optimal values are usually in the range from 0.02 to 1 mL/h [31].

The air pressure controls jet stretching and solvent evaporation. As the pressure increases, the fiber diameter and the number of structural defects decrease; however, excessively high pressure can cause jet instabilities due to Joule–Thomson effects. Studies have shown that an air-pressure range of 0.2–2.5 bar is optimal for investigating the influence of pressure on fiber formation [32].

Thus, in this work, the main process parameters were varied within the following ranges: solution flow rate—0.1, 0.2, 0.4, and 1 mL/h; air pressure—0.2, 0.6, 1.0, 1.5, 2.0, and 2.5 bar; polymer concentration—13, 14, and 15 wt.%.

The concentration range of 13–15 wt.% PAN in DMF was not chosen arbitrarily, but corresponds to the experimentally established spinnability window of the system in SBS. Preliminary experiments showed that below approximately 13 wt.% the solution does not provide sufficient chain entanglement, resulting in bead-on-string structures and jet break-up, whereas above 15 wt.% the solution viscosity becomes too high, leading to frequent nozzle clogging and unstable spinning.

The chosen ranges of polymer concentration, air pressure and solution flow rate provide a practical compromise between jet stability, suppression of bead-on-string defects and control of fiber diameter, which is consistent with previously reported observations for the SBS process.

### 2.5. Determination of Residual Solvent Content

The residual solvent content in the PAN nanofiber mats was determined by measuring the mass loss of the samples before and after drying at a controlled temperature. For each set of SBS processing conditions, three independent PAN nanofiber mats were produced.

Initial and final masses were measured on an analytical balance with a readability of 0.01 mg. The samples were dried in a convection oven at 100 °C for 30 min and then re-weighed under the same conditions.

Preliminary time-dependent drying tests were carried out on representative specimens to verify the adequacy of the chosen drying conditions: after the initial 30 min at 100 °C, samples were subjected to additional 30 min drying intervals with intermediate mass measurements. No statistically significant further mass loss was observed, indicating that a constant mass was reached under these conditions. Therefore, the values reported in this work should be interpreted as the residual volatile content under the standardized drying protocol, which is primarily used to analyze relative trends in solvent removal between different SBS process conditions.

### 2.6. SEM Analysis and Determination of Fiber Diameter

In addition to qualitative assessment of the obtained images, automated image analysis was performed using the General Image Fiber Tool (GIFT) method. The GIFT method has been successfully applied for computer analysis of images of fibrous structures [33].

Before computer analysis, the SEM images undergo preprocessing, which includes two steps:

1. Noise reduction. To eliminate imaging artifacts in the form of noise, median blurring and Gaussian blurring are applied.
2. Fiber segmentation. For visual separation of fibers from the background, automatic binarization is performed using Otsu’s method. This algorithm optimizes the threshold intensity level, thereby minimizing the variance between the background and the objects, which makes it possible to distinguish fibers even when the illumination in the image is non-uniform.

**Figure 4:**
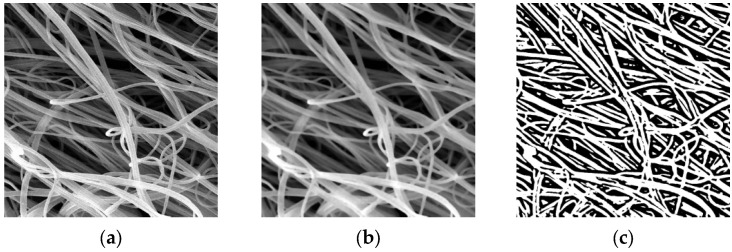
Image-processing steps for a SEM image of a fibrous structure: (**a**) original image; (**b**) blurred image; (**c**) automatic local binarization using Otsu’s method.

The image-processing steps are illustrated in Figure 4.

For each sample, a series of 100 measurements was carried out on different fibers in order to obtain statistically significant data. The number-average fiber diameter for each sample was calculated using Equation (1):

(1) $${\bar{d}}_{n}=\sum_{i}\frac{n_{i}}{\sum_{i}n_{i}}\times d_{i}=\sum_{i}f_{ni}d_{i},$$

where *nᵢ* is the number of fibers in the *i*-th size fraction (with diameter *dᵢ*), *N* is the total number of fibers in the sample, and *xᵢ* = *nᵢ*/*N* is the number fraction of the *i*-th size class. As a result of the computer analysis of the SEM images, fiber size distributions were obtained for each sample.

For each set of SBS processing conditions, fiber diameters were determined from multiple images acquired at the same magnification and, where necessary, at different magnifications from different positions on the nanofiber mat (and, for selected conditions, from independently prepared mats). Only fiber segments that appeared clearly in focus with well-defined edges were included in the automated analysis, whereas blurred or partially obscured fibers were excluded. This procedure mitigates depth-of-focus limitations and local inhomogeneities in the mat and improves the statistical representativeness of the calculated mean diameters.

### 2.7. Regression Analysis Method

In multiple regression, the model is usually represented in the following form (Equation (2)):

(2) $$Y=\beta_{0}+\beta_{0}X_{1}+\beta_{2}X_{2}+\cdots +\beta_{p}X_{p}$$

where *y* is the dependent variable, *x*_1_, *x*_2_, …, *xₚ* are the independent variables, and β_0_, β_1_, …, βₚ are the regression coefficients.

To estimate the coefficients β, the least squares method is used, which minimizes the sum of squared deviations of the observed values from the model predictions (Equation (3)):

(3) $$\underset{\beta }{\min}\sum_{i=1}^{n}{\left(y_{i}-\hat{y_{i}}\right)}^{2}=\underset{\beta }{\min}\sum_{i=1}^{n}{{(y}_{i}-\beta_{0}-\sum_{j=1}^{p}\beta_{j}x_{ij})}^{2}$$

The algorithm for computing the least-squares coefficients consists of the following steps:

1. Form the observation matrix X of size n × (p + 1), where n is the number of observations: the first column contains ones (the intercept term), and the remaining columns contain the values of the independent variables;
2. Form the vector Y of observed values of the dependent variable;
3. Compute the coefficient vector according to Equation (4):

(4) $$\hat{\beta }={\left(X^{T}X\right)}^{-1}X^{T}Y$$

where *Xᵀ* is the transpose of *X*, and (XᵀX)^−1^ is the inverse matrix, provided that it exists.

However, constructing the regression from a full factorial experiment is a special case that allows the normal equations to be represented in simplified form through scalar products (Equation (5)):

(5) $$b_{j}=\frac{\sum_{i=1}^{N}x_{ij}\times y_{i}}{N}$$

4. The obtained coefficients are then substituted into the regression equation for prediction and analysis.
5. The statistical significance of the regression coefficients is assessed using the Student *t*-test. The tabulated Student’s *t* value for the present model is 2.09.
6. The adequacy of the model is evaluated using the Fisher *F* test. The model is considered adequate if the calculated value of the *F* criterion is lower than the tabulated value for the selected significance level and degrees of freedom. For this model, the tabulated Fisher criterion at *p* = 0.05 is 2.6.

## 3. Results and Discussion

### 3.1. Nanofibers Structure

#### 3.1.1. Residual Solvent Content

The residual solvent content was measured by determining the mass loss of the nanofiber samples before and after drying in an oven at a controlled temperature (100 °C) and for a fixed time interval (30 min). The residual solvent content was calculated using Equation (6):

(6) $$W=\;\frac{m_{0}-\;m_{1}}{m_{0}}\times 100\%,$$

where W—residual solvent content, %; $m_{0}$—initial mass of the sample, g; $m_{1}$—mass of the sample after drying, g.

**Figure 5:**
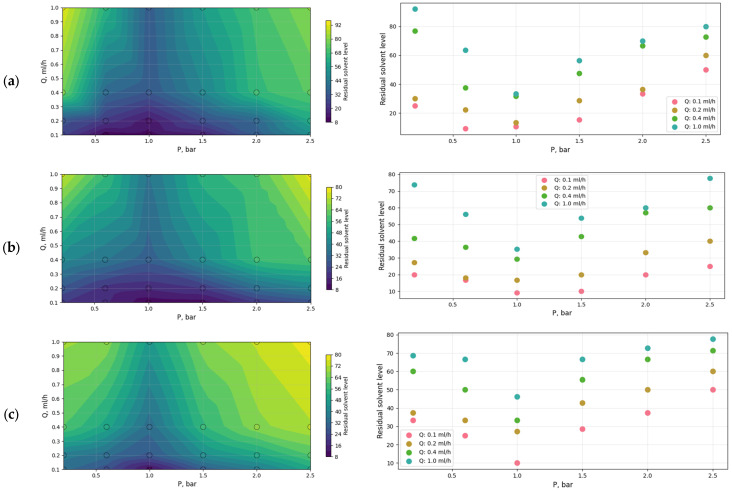
Visualization of the experimental results in the form of contour plots and dependences of the solution content on pressure at different flow rates for solutions with concentrations of: (**a**) 13 wt.%; (**b**) 14 wt.%; (**c**) 15 wt.%.

The results of the measurements for the obtained samples are presented in the Supplementary Materials (Tables S1–S3). Figure 5 provides a graphical visualization of the experimental data.

During nanofiber formation by solution blow spinning, the residual solvent content in the final material is controlled by heat and mass transfer processes. The Nusselt number (Nu) characterizes the intensity of convective heat transfer relative to conductive heat transfer and is defined as follows (Equation (7)):

(7) $$Nu=\;\frac{\alpha L}{\lambda },$$

where α—heat-transfer coefficient, L—characteristic length, λ—thermal conductivity of the medium.

The Sherwood number (Sh) (Equation (8)) describes an analogous balance for mass transfer, taking into account convection and diffusion:

(8) $$Sh=\;\frac{k_{c}L}{D},$$

where $k_{c}$—mass-transfer coefficient, L—characteristic length, D—diffusion coefficient of the solvent.

When the pressure of the compressed gas stream is increased from 0.2 to 1 bar, the gas velocity and the intensity of convective heat and mass transfer increase, which leads to higher Nu and Sh values and, consequently, to more efficient solvent removal from the fiber surface. This explains the decrease in residual solvent content in the material.

However, with a further increase in pressure, the opposite effect is observed. Excessively high gas velocities can induce flow instabilities and enhance turbulent mixing, which reduces the residence time of solvent molecules near the fiber surface and limits the efficiency of heat and mass transfer. This manifests itself as a decrease or stabilization of the Nu and Sh criteria.

In combination, a reduction in the solution flow rate additionally improves solvent removal by increasing the characteristic residence time of the solution in the high-velocity gas stream and by promoting more uniform stretching of the jet. This effect is reflected in changes in the Reynolds (Re), Prandtl (Pr), and Schmidt (Sc) numbers (Equation (9)):

(9) $$N_{u}=\;C_{1}{Re}^{m}{Pr}^{n},\;\;\;\;\;\;\;\;Sh=C_{2}{Re}^{p}{Pr}^{q},$$

where C_1_, C_2_, m, n, p, q are empirical constants that reflect the flow regime and the geometry of the system.

Under these conditions, the Reynolds number and, accordingly, the Nu and Sh criteria increase, which leads to intensification of heat and mass transfer, as confirmed by the decrease in residual solvent content.

Thus, in the present case, the optimal pressure range in the solution blow spinning process is 0.6–1.5 bar. Within this range, the efficiency of solvent removal is maximized owing to a balance between the mass- and heat-transfer criteria (Sh and Nu), the residence time of the material in the working zone, and the structural features of the flow. A further increase in pressure leads to a decrease in the efficiency of solvent removal from the jet, which accounts for the appearance of the observed minimum in solvent content. At the same time, the samples obtained at a solution flow rate of 1 mL/h contain a significantly higher residual solvent content. In this case, the optimal range of solution flow rate is 0.1–0.4 mL/h, which provides a balance between the rate of solvent evaporation and the formation of a stable nanofiber structure. To subsequently determine the diameter of the nanofibers obtained under these conditions, scanning electron microscopy images were obtained for these samples.

#### 3.1.2. Nanofibers Diameter

Based on the calculated number-average diameters (Supplementary Materials, Table S4), a dependence of nanofiber diameter on polymer concentration, air pressure, and solution flow rate was obtained. It was found that an increase in PAN concentration from 13 to 15 wt.% leads to a monotonic growth of the average nanofiber diameter. This behavior is associated with an increase in solution viscosity and elastic forces, which resist stretching. Under these conditions, the jet becomes more stable and less susceptible to thinning, which results in the formation of thicker fibers.

At the same time, an increase in air pressure within the studied range promotes more intensive stretching and thinning of the jet, leading to a decrease in fiber diameter. However, when the pressure exceeds a certain threshold, further increase is not accompanied by a noticeable reduction in diameter, since the energy of the gas stream becomes insufficiently efficiently converted into the stretching of a highly viscous solution and partly dissipates in turbulent vortices. A similar effect is observed at excessively high solution flow rates, when the volume of solution supplied to the nozzle exceeds the ability of the gas flow to efficiently break it up.

**Figure 6:**
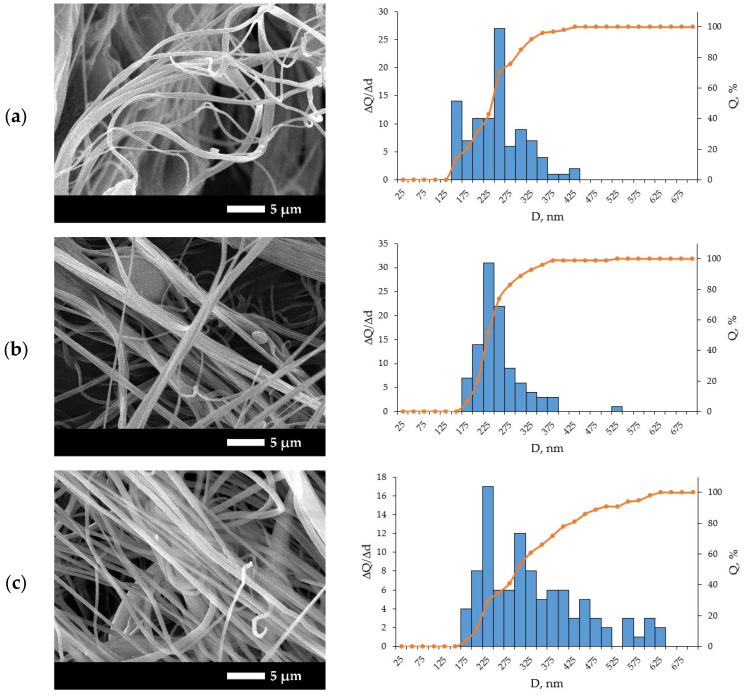
SEM images of the obtained nanofibers and histograms of their size distribution for samples produced at a pressure of 1 bar and a solution flow rate of 0.1 mL/h with different PAN concentrations: (**a**) 13 wt.%; (**b**) 14 wt.%; (**c**) 15 wt.%.

The SEM images obtained for the samples with the lowest residual solvent content and the corresponding fiber diameter histograms are shown in Figure 6 for different polymer concentrations.

The SEM images shown in Figure 6 should be regarded as representative examples of the morphology used for diameter extraction rather than the complete set of images used in the analysis. Each histogram exhibits a well-defined peak, which indicates a dominant fiber diameter with some scatter. The maximum of the distributions shifts toward larger diameters as the polymer concentration increases, which is consistent with the observed effect of solution viscosity. At the same time, as the technological parameters are optimized, the width of the distributions decreases, which reflects an improvement in fiber uniformity and a reduction in size variation.

### 3.2. Regression Analysis Results

As a result of the regression analysis, the following coefficients for the regression equation were obtained (Table 1).

After checking the significance of the parameters by the Student *t*-test, the terms whose coefficients were not statistically significant were excluded from the model. The final regression equation has the following form (Equation (10)):

(10) Y=300.48+37.19×C−16.59×P+16.26×Q

The Fisher criterion was calculated to assess the statistical significance of the regression model. The calculated value of the criterion is 0.39, which is lower than the critical tabulated value. Thus, the model is statistically significant.

To evaluate the quality of approximation of the experimental data by the regression model, the coefficient of determination *R*^2^ was used (Equation (11)):

(11) $$R^{2}=1-\frac{\sum {\left(y_{i}-\hat{y_{i}}\right)}^{2}}{\sum {\left(y_{i}-\bar{y}\right)}^{2}}$$

where *yᵢ* are the observed values, ŷᵢ are the values calculated by the model, and ȳ is the mean of the observed values.

In this study, the value of *R*^2^ was 0.85, which indicates a high degree of consistency between the model and the experimental data. It should be highlighted that the proposed regression equation is a local predictive model, valid within the experimentally investigated design space, i.e., PAN concentrations of 13–15 wt.%, air pressures of 0.2–2.5 bar, and solution flow rates of 0.1–1 mL h^−1^. The model is not intended to be extrapolated outside this SBS processing window, where the spinnability and fiber morphology can change qualitatively.

While design-of-experiments and regression models have been widely employed for electrospinning, analogous predictive tools for SBS—and particularly for PAN/DMF systems—are still comparatively scarce. The present model, therefore, complements existing SBS studies by providing a statistically validated, system-specific description of how the three key process parameters jointly affect PAN nanofiber diameter, in parallel with the analysis of residual solvent content.

## 4. Conclusions

A comprehensive investigation of the polyacrylonitrile (PAN) nanofiber production process by solution blow spinning showed that a reduction in solution flow rate combined with an increase in gas pressure to an optimal range leads to higher Reynolds (Re), Nusselt (Nu), and Sherwood (Sh) numbers, which reflect more efficient convective heat and mass transfer. This, in turn, ensures the formation of fibers with a minimal residual solvent content, thereby improving the quality of the material. When the pressure exceeds the optimal range, the gas velocity becomes excessively high, the contact time decreases, and the efficiency of mass transfer is reduced due to hydrodynamic limitations.

The study of the dependence of nanofiber diameter on the technological process parameters demonstrated that increasing the PAN concentration in the spinning solution results in a nearly linear increase in fiber diameter owing to the corresponding increase in viscosity. An increase in compressed gas pressure promotes a decrease in fiber diameter due to more intensive stretching of the polymer jet. In contrast, higher solution flow rates lead to the formation of larger droplets, which reduces the efficiency of forming thin fibers.

The regression analysis showed that the developed model, with a coefficient of determination R^2^ = 0.85, provides a qualitatively and quantitatively adequate description of the experimental data and explains a significant part of the variation in the diameters of the obtained nanofibers. The statistical significance of the effects of pressure and solution flow rate on fiber diameter was confirmed, which ensures the reliability of the model for predicting and optimizing the technological parameters of the process.

## Figures and Tables

**Table 1:** Coefficients of the regression equation.

Coefficients	b_0_	b_1_	b_2_	b_3_	b_4_	b_5_	b_6_
Values	300.48	37.19	−16.59	16.26	−2.29	4.11	−0.89

## Data Availability

The original contributions presented in this study are included in the article/Supplementary Materials. Further inquiries can be directed to the corresponding author.

## Supplementary Materials

The following supporting information can be downloaded at: https://www.mdpi.com/article/10.3390/polym18010100/s1, Table S1: Residual solvent level in samples (13 wt. %); Table S2: Residual solvent level in samples (14 wt. %); Table S3: Residual solvent level in samples (15 wt. %); Table S4: The calculated average diameter of the obtained nanofibers; Figure S1: SEM images of the obtained nanofibers and histograms of the diameter distribution for samples with a PAN concentration of 13 wt. %: (1)—pressure 0.6 bar, flow rate 0.4 mL/h; (2)—pressure 0.6 bar, flow rate 0.2 mL/h; (3)—pressure 0.6 bar, flow rate 0.1 mL/h; (4)—pressure 1 bar, flow rate 0.4 mL/h; (5)—pressure 1 bar, flow rate 0.2 mL/h; (6)—pressure 1 bar, flow rate 0.1 mL/h; (7)—pressure 1.5 bar, flow rate 0.4 mL/h; (8)—pressure 1.5 bar, flow rate 0.2 mL/h; (9)—pressure 1.5 bar, flow rate 0.1 mL/h; Figure S2: SEM images of the obtained nanofibers and histograms of the diameter distribution for samples with a PAN concentration of 14 wt. %: (1)—pressure 0.6 bar, flow rate 0.4 mL/h; (2)—pressure 0.6 bar, flow rate 0.2 mL/h; (3)—pressure 0.6 bar, flow rate 0.1 mL/h; (4)—pressure 1 bar, flow rate 0.4 mL/h; (5)—pressure 1 bar, flow rate 0.2 mL/h; (6)—pressure 1 bar, flow rate 0.1 mL/h; (7)—pressure 1.5 bar, flow rate 0.4 mL/h; (8)—pressure 1.5 bar, flow rate 0.2 mL/h; (9)—pressure 1.5 bar, flow rate 0.1 mL/h; Figure S3: SEM images of the obtained nanofibers and histograms of the diameter distribution for samples with a PAN concentration of 15 wt. %: (1)—pressure 0.6 bar, flow rate 0.4 mL/h; (2)—pressure 0.6 bar, flow rate 0.2 mL/h; (3)—pressure 0.6 bar, flow rate 0.1 mL/h; (4)—pressure 1 bar, flow rate 0.4 mL/h; (5)—pressure 1 bar, flow rate 0.2 mL/h; (6)—pressure 1 bar, flow rate 0.1 mL/h; (7)—pressure 1.5 bar, flow rate 0.4 mL/h; (8)—pressure 1.5 bar, flow rate 0.2 mL/h; (9)—pressure 1.5 bar, flow rate 0.1 mL/h.
