# Supplementary material for: Polyacrylonitrile Nanofiber Mats Produced by Solution Blow Spinning: Influence of Process Parameters on Fiber Diameter and Residual Solvent Content
Source: Polymers (Basel). 2025 Dec 29;18(1):100. doi: 10.3390/polym18010100 (PMC12787849; doi:10.3390/polym18010100)
Supplement: Supplementary file 1 [file polymers-18-00100-s001.zip › polymers-4034743-supplementary.pdf]

Table S1. Residual solvent level in samples (13 wt. %).

№	P, bar	Q, ml/h	W, %
1	0.2	0.1	25±2
2		0.2	30±8
3		0.4	77±7
4		1	92±10
5	0.6	0.1	9±4
6		0.2	22±3
7		0.4	38±12
8		1	64±9
9	1	0.1	11±1
10		0.2	13±3
11		0.4	312±2
12		1	33±2
13	1.5	0.1	15±2
14		0.2	29±11
15		0.4	48±3
16		1	56±6
17	2	0.1	33±5
18		0.2	36±5
19		0.4	67±2
20		1	70±3
21	2.5	0.1	50±3
22		0.2	60±5
23		0.4	73±9
24		1	80±10

Table S2. Residual solvent level in samples (14 wt. %).

№	P, bar	Q, ml/h	W, %
1	0.2	0.1	20±2
2		0.2	27±5
3		0.4	42±11
4		1	74±15
5	0.6	0.1	17±1
6		0.2	18±2
7		0.4	36±8
8		1	56±11
9	1	0.1	9±3
10		0.2	17±4
11		0.4	29±8
12		1	35±5
13	1.5	0.1	10±2
14		0.2	20±7
15		0.4	43±4
16		1	54±9
17	2	0.1	20±3
18		0.2	33±8
19		0.4	57±5
20		1	60±3
21	2.5	0.1	25±5
22		0.2	40±6
23		0.4	60±10
24		1	78±7

Table S3. Residual solvent level in samples (15 wt. %).

№	P, bar	Q, ml/h	W, %
1	0.2	0.1	33±2
2		0.2	38±2
3		0.4	60±10
4		1	69±6
5	0.6	0.1	25±1
6		0.2	33±8
7		0.4	50±5
8		1	67±10
9	1	0.1	10±5
10		0.2	27±2
11		0.4	33±3
12		1	46±6
13	1.5	0.1	29±8
14		0.2	43±2
15		0.4	56±5
16		1	67±6
17	2	0.1	38±3
18		0.2	50±6
19		0.4	67±3
20		1	73±6
21	2.5	0.1	50±2
22		0.2	60±5
23		0.4	71±3
24		1	78±3

Table S4. The calculated average diameter of the obtained nanofibers.

№	C, wt. %	P, bar	Q, ml/h	D, nm
5	13	0.6	0.1	255
6			0.2	263
7			0.4	280
9		1	0.1	244
10			0.2	253
11			0.4	260
13		1.5	0.1	221
14			0.2	239
15			0.4	248
29	14	0.6	0.1	280
30			0.2	342
31			0.4	353
33		1	0.1	248
34			0.2	270
35			0.4	321
37		1.5	0.1	231
38			0.2	261
39			0.4	277
53	15	0.6	0.1	366
54			0.2	383
55			0.4	428
57		1	0.1	331
58			0.2	349
59			0.4	385
61		1.5	0.1	312
62			0.2	338
63			0.4	375

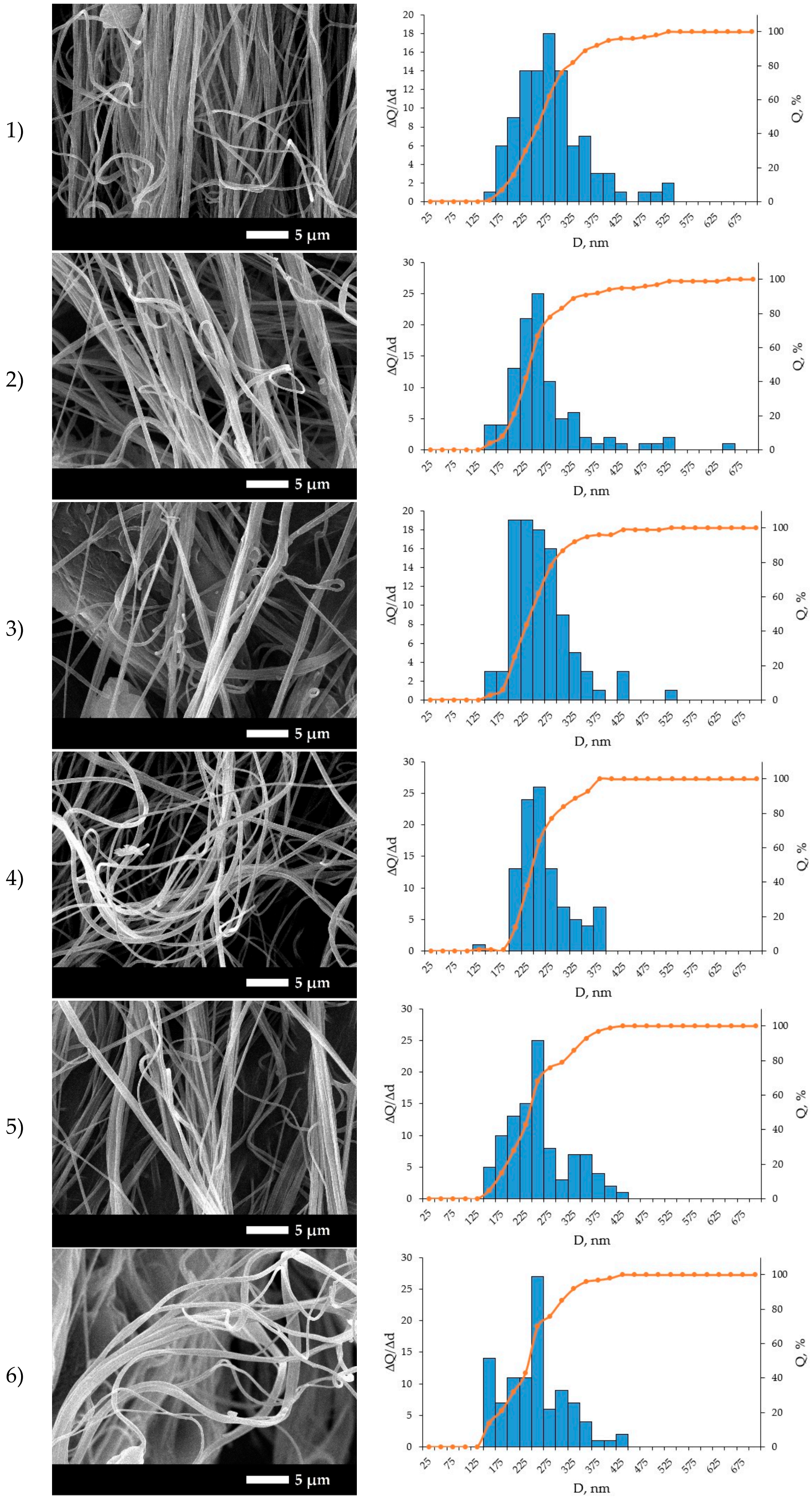

1)
2)
3)
4)
5)
6)
5 µm
ΔQ/Δd
Q, %
D, nm

7)

8)

9)

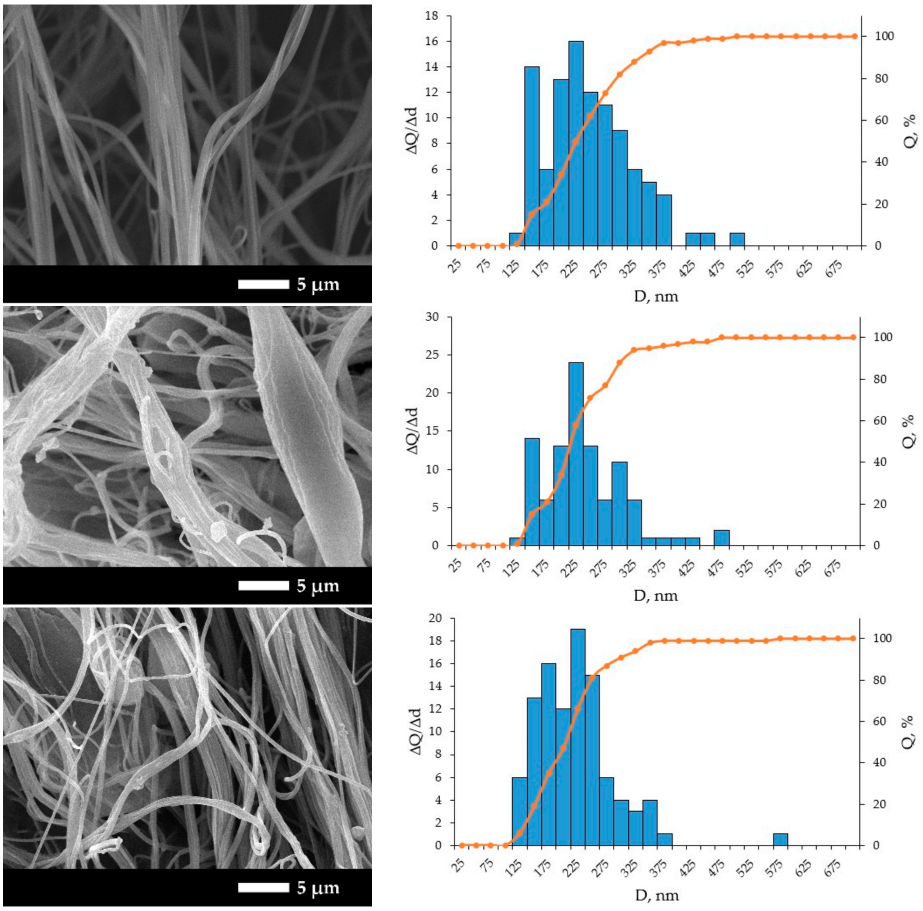

Figure S1. SEM images of the obtained nanofibers and histograms of the diameter distribution for samples with a PAN concentration of 13 wt. %: (1) – pressure 0.6 bar, flow rate 0.4 ml/h; (2) – pressure 0.6 bar, flow rate 0.2 ml/h; (3) – pressure 0.6 bar, flow rate 0.1 ml/h; (4) – pressure 1 bar, flow rate 0.4 ml/h; (5) – pressure 1 bar, flow rate 0.2 ml/h; (6) – pressure 1 bar, flow rate 0.1 ml/h; (7) – pressure 1.5 bar, flow rate 0.4 ml/h; (8) – pressure 1.5 bar, flow rate 0.2 ml/h; (9) – pressure 1.5 bar, flow rate 0.1 ml/h.

1)

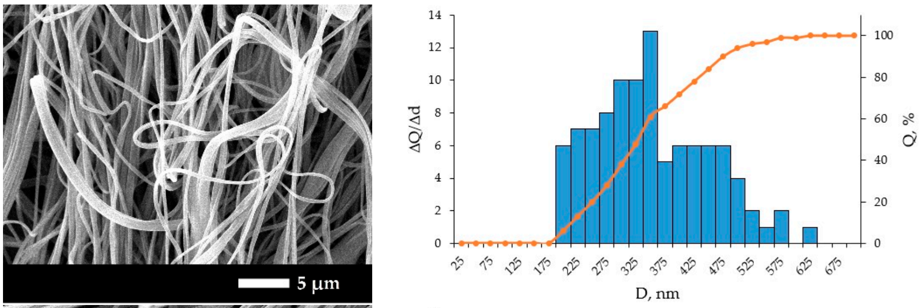
5 µm
ΔQ/Δd
Q, %
D, nm

2)

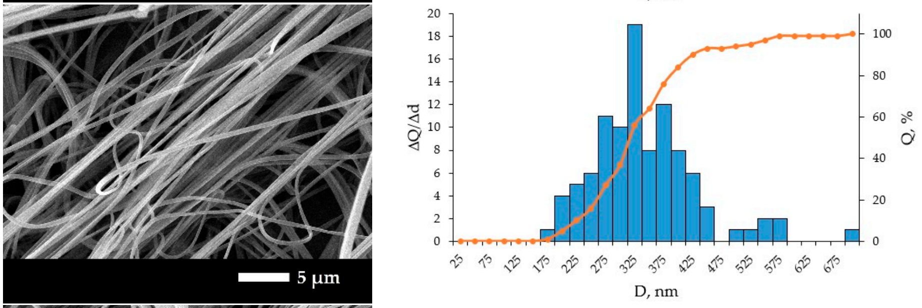
5 µm
ΔQ/Δd
Q, %
D, nm

3)

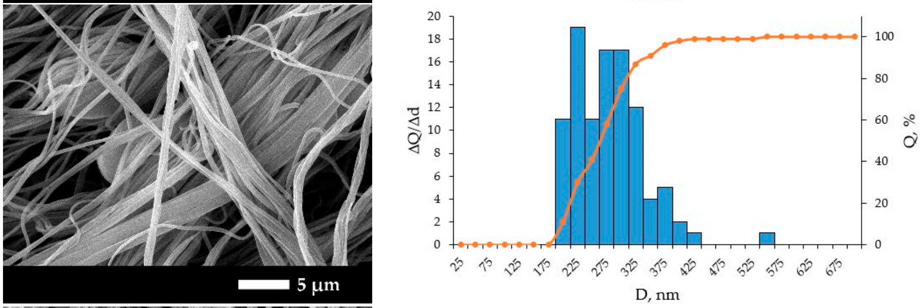
5 µm
ΔQ/Δd
Q, %
D, nm

4)

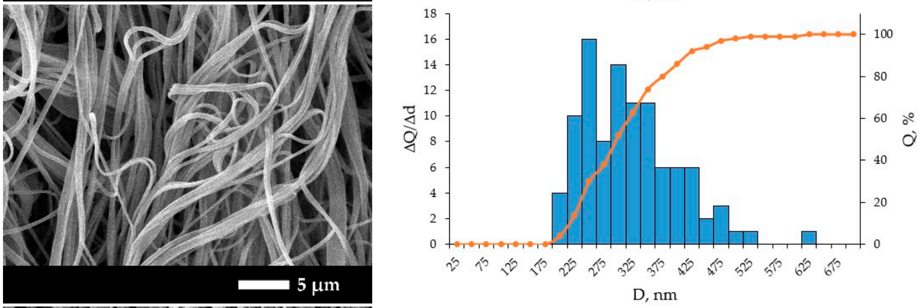
5 µm
ΔQ/Δd
Q, %
D, nm

5)

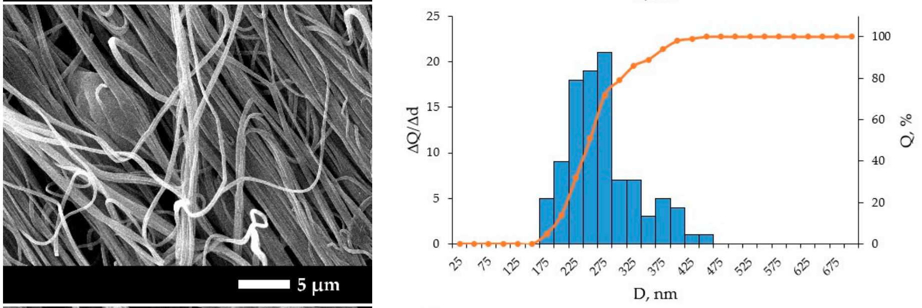
5 µm
ΔQ/Δd
Q, %
D, nm

6)

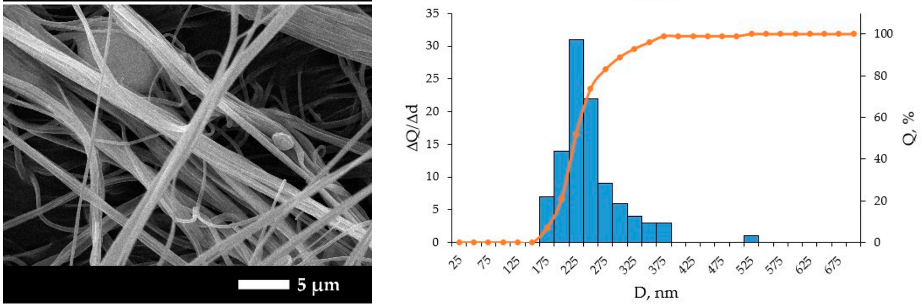
5 µm
ΔQ/Δd
Q, %
D, nm

7)

8)

9)

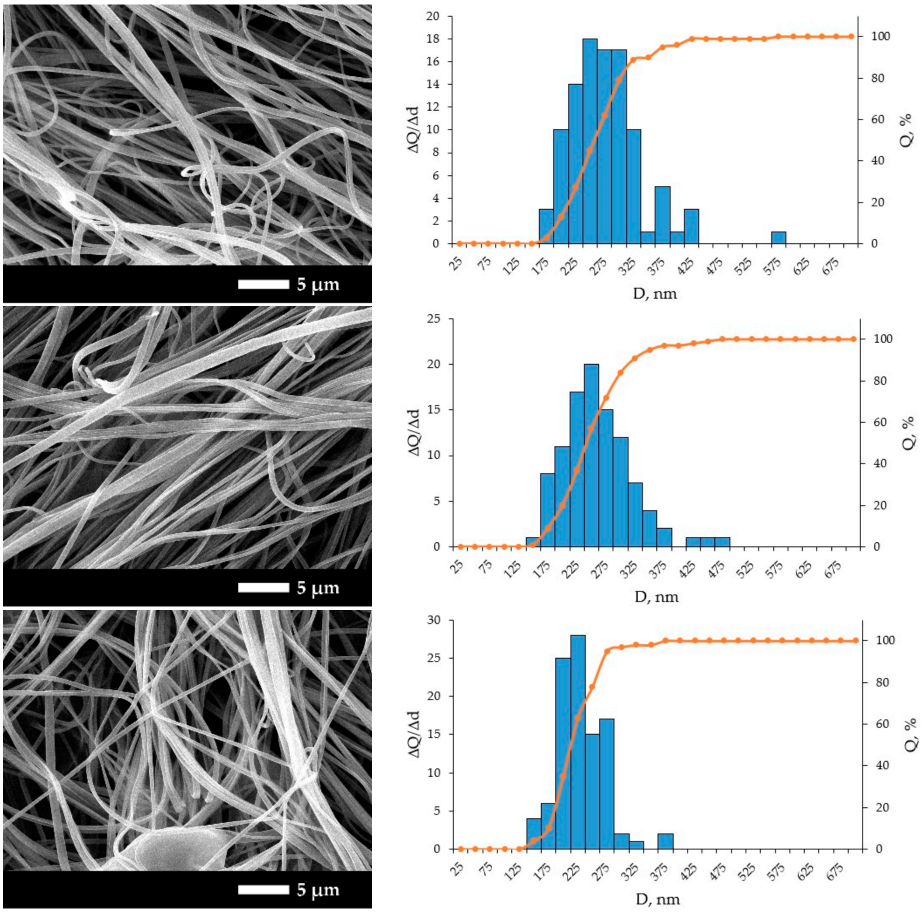

Figure S2. SEM images of the obtained nanofibers and histograms of the diameter distribution for samples with a PAN concentration of 14 wt. %: (1) – pressure 0.6 bar, flow rate 0.4 ml/h; (2) – pressure 0.6 bar, flow rate 0.2 ml/h; (3) – pressure 0.6 bar, flow rate 0.1 ml/h; (4) – pressure 1 bar, flow rate 0.4 ml/h; (5) – pressure 1 bar, flow rate 0.2 ml/h; (6) – pressure 1 bar, flow rate 0.1 ml/h; (7) – pressure 1.5 bar, flow rate 0.4 ml/h; (8) – pressure 1.5 bar, flow rate 0.2 ml/h; (9) – pressure 1.5 bar, flow rate 0.1 ml/h.

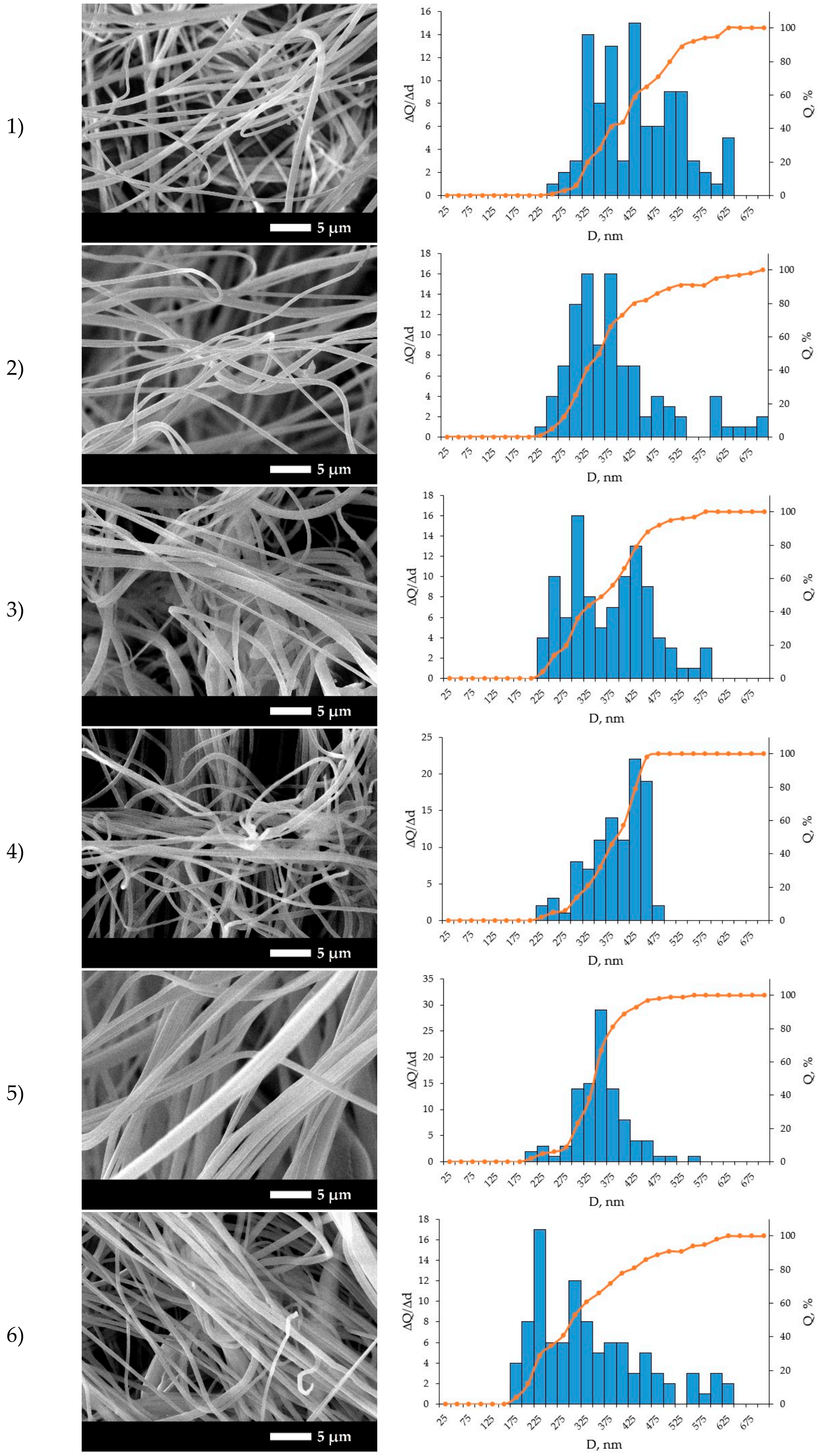

1)
2)
3)
4)
5)
6)
5 μm
ΔQ/Δd
Q, %
D, nm

7)

8)

9)

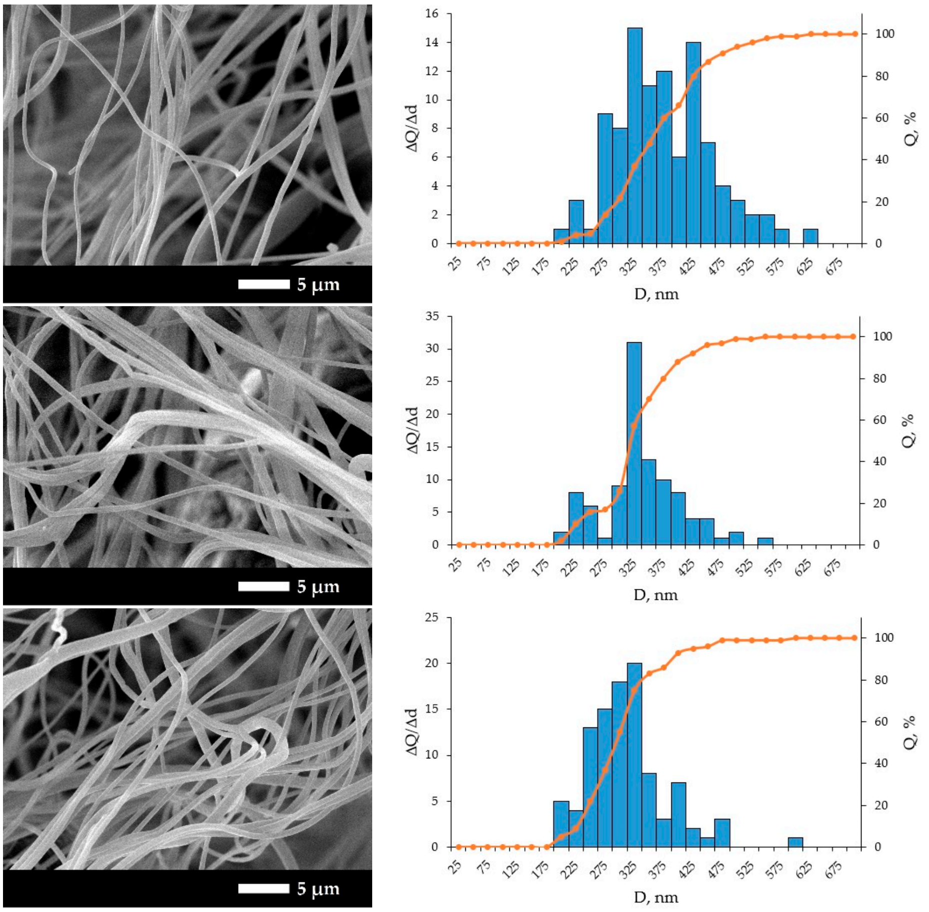

Figure S3. SEM images of the obtained nanofibers and histograms of the diameter distribution for samples with a PAN concentration of 15 wt. %: (1) – pressure 0.6 bar, flow rate 0.4 ml/h; (2) – pressure 0.6 bar, flow rate 0.2 ml/h; (3) – pressure 0.6 bar, flow rate 0.1 ml/h; (4) – pressure 1 bar, flow rate 0.4 ml/h; (5) – pressure 1 bar, flow rate 0.2 ml/h; (6) – pressure 1 bar, flow rate 0.1 ml/h; (7) – pressure 1.5 bar, flow rate 0.4 ml/h; (8) – pressure 1.5 bar, flow rate 0.2 ml/h; (9) – pressure 1.5 bar, flow rate 0.1 ml/h.
